# Exploring the Dynamics of Perceived Organizational Support, Psychological Safety, and Organizational Justice in Enhancing Nurses’ Meaningful Work: A Moderated Mediation Model

**DOI:** 10.1155/nrp/3204260

**Published:** 2026-05-13

**Authors:** Ibrahim Abdullatif Ibrahim

**Affiliations:** ^1^ Department of Nursing Sciences, College of Applied Medical Sciences, Shaqra University, Shaqra, Saudi Arabia, su.edu.sa; ^2^ Department of Nursing Administration, Faculty of Nursing, Mansoura University, Mansoura, Egypt, mans.edu.eg

**Keywords:** job satisfaction, nurses, psychological safety, work engagement, working conditions

## Abstract

**Background:**

Meaningful work is a critical psychological resource that enhances nurse engagement, retention, and quality of patient care. Organizational factors, including perceived organizational support, psychological safety, and organizational justice, play key roles in shaping nurses’ perceptions of meaningful work. While previous studies have examined these factors individually, limited evidence exists on how they interact.

**Aim:**

To explore the relationships between perceived organizational support, psychological safety, and organizational justice in enhancing nurses’ meaningful work.

**Methods:**

This multicenter cross‐sectional study surveyed a convenience sample of 349 nurses from four general hospitals. Data were collected using online survey, and partial least squares approach was employed to examine the hypothesized relationships.

**Results:**

Perceived organizational support was positively associated with meaningful work (*β* = 0.421, *p* < 0.001) and psychological safety (*β* = 0.319, *p* < 0.001), whereas organizational justice was positively associated with psychological safety (*β* = 0.287, *p* < 0.001). Psychological safety was positively associated with meaningful work (*β* = 0.199, *p* < 0.01) and partially mediated the associations of perceived organizational support (*β* = 0.064, *p* < 0.05) and organizational justice (*β* = 0.057, *p* < 0.05) with meaningful work. Moderated mediation analysis revealed a small but statistically significant negative effect of organizational justice on the association between perceived organizational support and psychological safety (*f* ^2^ = 0.015), and the indirect association of perceived organizational support with meaningful work via psychological safety was weaker at higher levels of organizational justice.

**Conclusion:**

Meaningful work among nurses is supported by organizational support and fairness through psychological safety. High organizational justice may reduce reliance on perceived support. Nurse leaders should foster fair, supportive environments and policies that promote psychological safety, recognition, and engagement, ultimately enhancing retention, well‐being, and patient care.

## 1. Introduction

The quality and sustainability of healthcare systems increasingly depend on the psychological and professional well‐being of nurses, who are the frontline of patient care [1, 2]. As healthcare organizations face rising demands, high turnover rates, and growing emotional labor among nursing staff, there is mounting concern about how to cultivate a work environment that enables nurses not only to perform but also to flourish [3–5]. One of the most compelling yet under investigated outcomes in this regard is meaningful work; it is associated with higher job satisfaction, enhanced engagement, and improved care outcomes [6, 7]. However, what makes work meaningful for nurses, and how organizational factors contribute to this experience, remain insufficiently understood. Recent studies have emphasized the substantial influence of organizational characteristics on nurses’ perceptions of meaningful work. Perceived organizational support was linked with job satisfaction and overall well‐being among nurses [8]. Furthermore, psychological safety fosters higher work engagement and job satisfaction, reduces turnover intentions, and increases the likelihood of reporting patient safety concerns [2, 9]. Organizational justice also significantly impacts registered nurses′ work‐related outcomes, health, and well‐being [1, 10, 11].

Although much of the existing evidence derives from Western and East Asian contexts, Arab healthcare settings exhibit distinct cultural and organizational characteristics that may shape how nurses perceive support, fairness, and psychological safety. Research indicates that organizational justice in Arab contexts is strongly influenced by relational and interpersonal dynamics, with justice dimensions reflecting culturally embedded social norms [12, 13]. Additionally, hierarchical structures and high power distance in healthcare organizations can constrain communication and limit psychological safety, particularly in environments where deference to authority and fear of blame persist [14, 15]. Furthermore, collectivist and religiously informed cultural values play a central role in shaping workplace perceptions, where social support and normative endorsement significantly influence nurses’ attitudes and commitment [16]. Even when psychological safety is fostered, it remains embedded within hierarchical and collectivist norms, relying on culturally adapted leadership practices and fairness signals [17].

Despite the recognized importance of these organizational factors, there is a scarcity of research examining the integrated effect of perceived organizational support, psychological safety, and organizational justice on nurses’ experience of meaningful work. Most existing studies have explored these variables individually, lacking a comprehensive framework that captures their interrelationships and combined impact on meaningful work. Furthermore, much of the literature has originated from Western or East Asian contexts, leaving a significant cultural and contextual gap in understanding how these organizational factors work in the healthcare system. Accordingly, this study aims to address these gaps by investigating the influence of perceived organizational support on meaningful work, with psychological safety as a mediator and organizational justice as a moderator.

This study integrates four complementary theoretical perspectives to explain the mechanisms linking organizational context to meaningful work. First, Social Exchange Theory [18] posits that when nurses perceive organizational support and fairness, they reciprocate with trust, openness, and discretionary effort, foundations of psychological safety. Second, Self‐Determination Theory (SDT; [19]) suggests that meaningful work arises when basic psychological needs are met (autonomy, relatedness, and competence). Psychological safety supports these needs by enabling authentic expression and relational connection. Third, the Job Demands–Resources (JD‐R) model [20] frames perceived organizational support and organizational justice as job resources that buffer stress and enhance motivation. Finally, Conservation of Resources (COR) theory [21] helps interpret the moderation effect: when core resources like justice are depleted, employees rely more heavily on relational resources such as support. This integrated framework justifies testing psychological safety as a mediator and organizational justice as a moderator. Therefore, this study seeks to explore the dynamic relationships between perceived organizational support, psychological safety, and organizational justice in enhancing the nurses’ perception of meaningful work. Specifically, it focuses on the indirect mediating role of psychological safety and the moderating role of organizational justice in these relationships.

### 1.1. Literature Review and Hypotheses Development

#### 1.1.1. Perceived Organizational Support in Nursing

Perceived organizational support reflects employees′ perceptions that their organization appreciates their efforts and is concerned with their well‐being [22]. In the nursing environments characterized by high emotional and cognitive demands, this support is not merely appreciated, it is essential [3, 23]. Perceived organizational support communicates professional recognition and reinforces the idea that nursing work contributes meaningfully to the organization’s mission, particularly in delivering ethical and high‐quality care. When organizational support is perceived as authentic and consistent, it facilitates value congruence between nurses’ personal identities and their institutional role, thereby fostering greater perceptions of meaningful work [8].

#### 1.1.2. Perceived Organizational Support and Meaningful Work

Meaningful work is increasingly recognized as a multidimensional construct, encompassing a sense of purpose, alignment with personal identity, and the perceived significance of nurses’ tasks [24]. In the nursing context, it has been empirically associated with enhanced job satisfaction, performance, resilience, and reduced burnout [6, 7, 25]. Nurses who perceive their work as meaningful are more likely to demonstrate higher engagement and adaptability in stressful clinical contexts [26]. Understanding the drivers of meaningful work is essential for improving retention and professional fulfillment. Prior studies have found a consistent positive association between perceived organizational support and meaningful work. A supportive organizational climate enhances employees’ commitment, satisfaction, motivation, and psychological well‐being while mitigating stress [27, 28]. Drawing on the JD‐R model [29], perceived organizational support can be conceptualized as a job resource that buffers demand and enhances motivational pathways, thereby promoting the experience of work as meaningful. Therefore, the following hypothesis is proposed:

 H1: Perceived organizational support positively influences nurses’ perception of meaningful work.

#### 1.1.3. The Mediating Role of Psychological Safety

Psychological safety reflects the shared belief that individuals can express themselves without fear of embarrassment, punishment, or career‐related negative consequences [30]. In nursing, where high‐stakes decisions and collaborative care are routine, psychological safety is foundational to team learning, interpersonal openness, and adaptive behavior. Recent studies underscore its protective role in reducing occupational burnout and promoting work engagement, ultimately enhancing the psychological well‐being of nurses [2, 31, 32].

Perceived organizational support is a critical antecedent to psychological safety. When institutions demonstrate fairness, responsiveness, and concern for staff, they engender an environment of interpersonal trust and respect [33]. This relational environment further supportive supports the emergence of psychological safety by signaling that voicing concerns or making mistakes will not result in negative consequences [8, 34]. SET [18], which holds that relationships inside organizations are molded by norms of reciprocity, helps to clarify the link between perceived organizational support and psychological safety. Staff members are more likely to respond with actions that help the group when an organization indicates its commitment to employee well‐being, such as openness, teamwork, and discretionary effort. Psychological safety in this situation is a reciprocated reaction to organizational concern that lowers the perceived dangers of interpersonal exposure and promotes communicative openness.

Psychological safety is also a mechanism through which organizational support influences the experience of meaningful work. Psychological safety significantly enhances healthcare workers’ role involvement, which is a critical antecedent of perceiving work as meaningful [35]. Additionally, psychologically safe nursing environments are associated with improved patient safety outcomes, reduced turnover intentions, and greater job satisfaction [2]. Collectively, these factors contribute to stronger perceptions of meaningful work.

The relationship between psychological safety and meaningful work is grounded in SDT, which posits that meaningful work arises from the satisfaction of three basic psychological needs including competence, autonomy, and relatedness [19]. Extending SDT, [36] found that autonomy, relatedness, and beneficence were consistently and independently associated with perceived meaningful work, while competence showed a more variable effect. Psychological safety directly facilitates these conditions by promoting authentic self‐expression, fostering constructive feedback, and enabling relational connectedness. Thus, psychological safety is not only an outcome of perceived organizational support but also a mediator linking supportive organizational climates with meaningful work. Based on the foregoing theoretical and empirical evidence, the following hypotheses are proposed:

 H2: Perceived organizational support positively influences nurses’ perception of psychological safety.
 H3: Psychological safety positively influences nurses’ perception of meaningful work.
 H4: Psychological safety mediates the relationship between perceived organizational support and meaningful work.

#### 1.1.4. The Moderating Role of Organizational Justice

Organizational justice refers to the employees’ perceptions of fairness across three dimensions: procedural (fair decision‐making), distributive (equality in outcomes), and interactional (respectful treatment) [37]. In healthcare settings, these perceptions are instrumental in shaping how employees interpret organizational support and whether they trust leadership intentions [10, 38]. Beyond fostering trust, organizational justice represents a vital job resource within the JD‐R framework, capable of promoting motivational and psychological outcomes. Specifically, nurses who perceive high organizational justice are more likely to feel safe to voice concerns, take interpersonal risks, and fully engage in their work, thereby directly enhancing psychological safety. Recent empirical studies in nursing reinforce this relationship. For example, perceived organizational justice has been shown to improve new nurses’ work performance through mediators such as professional identity and work readiness [39], enhance job performance via organizational climate and job embeddedness [40], and reduce workplace deviant behavior through emotional labor and psychological capital [41].

Evidence further suggests that organizational justice may moderate the relationship between perceived organizational support and psychological safety. Previous research conducted in Taiwan found that nurses’ perceptions of organizational justice positively moderated the relationship between organizational support and their engagement in behaviors beneficial to the organization [42]. While this finding supports the interactive role of justice, the present study proposes a different directional mechanism grounded in the resource substitution logic of the JD‐R model and the resource scarcity principle of COR theory [21, 29]. Specifically, when structural resources such as fairness in procedures, interactions, and outcomes are abundant, additional relational resources, such as organizational support, may provide diminishing incremental value in fostering psychological safety. Conversely, in contexts where justice is perceived as low, perceived organizational support may become particularly salient, compensating for the lack of structural fairness and reinforcing nurses’ confidence in taking interpersonal risks.

This reasoning is further supported by empirical evidence from resource‐based studies. For instance, Venz et al. [43] demonstrated that employees’ use of compensatory strategies enhanced daily work engagement by offsetting low levels of job resources such as role clarity, job control, and recovery. Similarly, Xanthopoulou et al. [44] showed that job and personal resources are reciprocally related over time, with resources mutually substituting when others are scarce. Analogously, perceived organizational support may be less influential in highly just organizational environments but more critical when justice is perceived as low. This provides both theoretical and empirical justification for the hypothesized negative moderation effect.

Furthermore, leaders with high psychological capital may play a particularly important role in low‐justice environments [45], where supportive behaviors can compensate for perceived deficiencies in fairness and strengthen employees’ psychological security. Building on this reasoning, the present research proposes a moderated mediation model in which organizational justice not only directly influences meaningful work but also moderates the indirect effect of perceived organizational support on meaningful work through psychological safety.

Considering the preceding theoretical and empirical evidence, the following hypotheses are posited:

 H5: Organizational justice positively influences nurses’ perception of psychological safety.
 H6: Organizational justice negatively moderates the relationship between perceived organizational support and psychological safety.
 H7: The indirect effect of perceived organizational support on meaningful work via psychological safety is negatively moderated by organizational justice.

**FIGURE 1:**
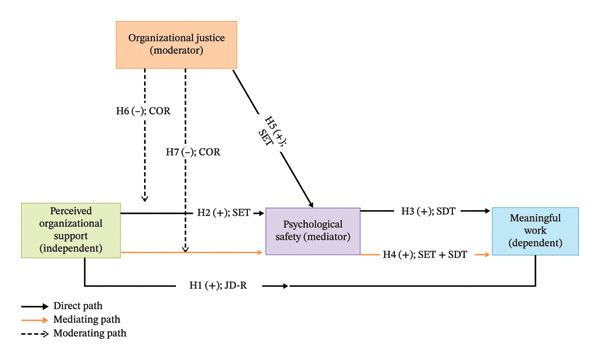
An integrative conceptual framework of the study. Note: SET = social exchange theory; SDT = self‐determination theory; JD‐R = job demands–resources model; COR = conservation of resources theory.

Based on the reviewed literature, hypotheses, and theoretical integration, an integrative conceptual framework guiding this study is presented in Figure 1.

## 2. Methods

### 2.1. Study Design and Sample

A multicenter cross‐sectional design was employed in accordance with the Strengthening the Reporting of Observational Studies in Epidemiology (STROBE) guidelines. The study was conducted across four general hospitals affiliated with the Riyadh Second Health Cluster in the Riyadh region of Saudi Arabia. These hospitals are public sector institutions that provide comprehensive healthcare services, including medical, surgical, emergency, and critical care, serving a diverse patient population. A convenience sampling strategy was used to recruit participants from various clinical departments (e.g., medical, surgical, and critical care units) to capture a broad range of nursing experiences. The study included 349 nursing staff (clinical nurses and nurse managers) who voluntarily agreed to participate, had at least one year of nursing experience, and were employed full time. Nursing staff from other professions (e.g., radiology, laboratory, pharmacy), part‐time nurses, and internship students were excluded.

The sample size for this study was determined based on the requirements of partial least squares structural equation modeling (PLS‐SEM), which is well‐suited for complex models with moderation effects and performs robustly even with moderate sample sizes. The minimum required sample was evaluated using the “10‐times rule,” which recommends that the sample size should be at least 10 times the largest number of indicators measuring any single construct or the largest number of structural paths directed at an endogenous construct [46]. In this study, the four constructs comprised 20 indicators, resulting in a minimum required sample of 200 participants. Ultimately, 349 participants were recruited due to the availability of additional eligible respondents, substantially exceeding the PLS‐SEM threshold. This sample size ensures reliable estimation of both direct and conditional effects.

### 2.2. Measures and the Study Variables

The study variables included four variables and demographics:

Perceived organizational support: measured using the shortened version of the perceived organizational support scale [22], comprising four positive and four negative items (reverse‐coded) on a 7‐point Likert scale (0 = strongly disagree, 6 = strongly agree).

Psychological safety: assessed using a 5‐item scale [47] rated on a 5‐point scale from 1 (not at all) to 5 (a large extent).

Organizational justice: measured using a 4‐item scale [1], with responses on a 5‐point scale ranging from 1 (to a very small extent) to 5 (to a very large extent).

Meaningful work: evaluated using a 3‐item scale adapted from the psychological empowerment scale [48], with responses on a 5‐point Likert scale from 1 (strongly disagree) to 5 (strongly agree).

Demographics: age, gender, marital status, education, position, nationality, and years of experience were collected.

All scales were translated from English into Arabic and back‐translated using Brislin’s guidelines [49]. A panel of three nursing faculty members assessed semantic and conceptual equivalence, achieving a Content Validity Index (CVI) of 0.92. A pilot study with 27 nurses ensured clarity and identified potential response biases; no major issues were reported. Pilot participants were not included in the main sample. The internal consistency reliability of the core scales was acceptable, with Cronbach’s α values of 0.85 for perceived organizational support, 0.79 for psychological safety, 0.82 for organizational justice, and 0.81 for meaningful work, supporting their use in the Saudi nursing context.

### 2.3. Data Collection

Data were gathered via an online survey using Google Forms from January to March 2025. Before participating in the study, all participants were required to provide electronic consent. Participants were given an information and consent form to review before they could begin the online questionnaire. The form explained the research’s goals, methods, and participants′ rights. Participants had to press the button “I agree” to show their consent. It was mandatory to provide consent to proceed to the questionnaire; those who did not agree were redirected to an exit page. To ensure data completeness, all responses in the questionnaire were set as mandatory, preventing missing data. Reminder emails were sent to the participants 2 weeks after the initial contact.

### 2.4. Statistical Analysis

Data were analyzed using the PLS_SEM approach via Smart‐PLS 4.1.1.2 and SPSS version 27. The study was conducted in two phases: evaluation of the measurement model and assessment of the structural model. Direct, indirect, and moderated mediation effects were assessed for significance using bootstrapping with 5000 resamples. Descriptive statistics, including means, standard deviations, and frequencies, were calculated to describe demographic information and research variables. Harman’s single‐factor test was conducted to evaluate possible common method bias, indicating that a single component explained 38.8% of the total variance, which is below the recommended threshold of 50%, thus demonstrating that common bias is not a significant issue [50].

### 2.5. Ethical Considerations

Ethical approval was obtained from the Research Ethics Committee at Shaqra University (Approval No: ERC_SU_S_202500021), and the study adhered to the principles of the Declaration of Helsinki. Prior to participation, individuals were presented with an electronic informed consent form that outlined the study’s aims, procedures, voluntary nature of participation, and participants’ rights, including the right to withdraw at any time without penalty.

## 3. Results

### 3.1. Characteristics of the Sample

Out of the 390 questionnaires mailed, 349 completed (89.5%. response rate). The remaining 41 responses consisted of 24 refusals and 17 exclusions due to not meeting the inclusion criteria.

The majority of the participants were aged between 31 and 40 years (73.6%), followed by those aged 20–30 years (20.1%), and those over 40 years (6.3%). The majority of respondents were female (83.4%) and married (87.1%). Concerning educational qualifications, 57.0% had a bachelor’s degree, 38.1% held a technical certification, and 4.9% had attained postgraduate education. Regarding job experience, 39.3% had more than 10 years, 38.4% had 6–10 years, and 22.3% had 1–5 years of experience. Most participants were employed as staff nurses (94.6%), while a minor percentage held management positions (5.4%) (Table 1).

**TABLE 1:** Characteristics of the participants.

Characteristics	*N*	%
*Age (years)*		
20–30	70	20.1
31–40	257	73.6
> 40	22	6.3

*Gender*		
Female	291	83.4
Male	58	16.6

*Marital status*		
Unmarried	45	12.9
Married	304	87.1

*Education*		
Technical degree	133	38.1
Bachelor degree	199	57.0
Postgraduate studies	17	4.9

*Experience*		
1–5	78	22.3
6–10	134	38.4
> 10	137	39.3

*Nursing category*		
Staff nurse	330	94.6
Nursing managers	19	5.4

### 3.2. Measurement Model Evaluation

The reliability and validity of the reflective measurement model were assessed by indicator loadings, internal consistency reliability, convergent validity, and discriminant validity. Regarding indicator reliability, all indicators outer loadings surpassed the recommended threshold of 0.708 [51], ranging from 0.710 (POS8) to 0.886 (MW2), indicating acceptable item reliability. Regarding internal consistency, Cronbach’s alpha (α) and composite reliability (CR) values for all constructs surpassed the acceptable threshold of 0.70 [51]. Specifically, CR values were 0.920 (POS), 0.868 (PS), 0.895 (OJ), and 0.897 (MW), confirming satisfactory internal consistency (Table 2).

**TABLE 2:** The descriptive statistics, reliability, and convergent validity of the constructs.

Constructs	Items	Loading	VIF	*α*	CR	AVE	Mean (SD)
Perceived Organizational support (POS)	POS1	0.716	1.878	0.900	0.920	0.590	3.85 (0.95)
	POS2	0.772	2.165				
	POS3	0.822	2.570				
	POS4	0.795	2.230				
	POS5	0.781	2.273				
	POS6	0.783	2.389				
	POS7	0.758	2.069				
	POS8	0.710	1.816				

Psychological Safety (PS)	PS1	0.722	1.481	0.810	0.868	0.569	4.02 (0.63)
	PS2	0.791	1.690				
	PS3	0.752	1.534				
	PS4	0.778	1.673				
	PS5	0.726	1.530				

Organizational justice (OJ)	OJ1	0.800	1.975	0.844	0.895	0.681	3.87 (0.84)
	OJ2	0.833	2.241				
	OJ3	0.848	1.995				
	OJ4	0.799	1.684				

Meaningful work (MW)	MW1	0.838	1.757	0.828	0.897	0.744	4.03 (0.72)
	MW2	0.886	2.048				
	MW3	0.863	1.931				

Regarding convergent validity, the average variance extracted (AVE) values ranged from 0.569 to 0.744, all above the threshold of 0.50 [51], confirming convergent validity.

The Fornell–Larcker criterion, cross‐loadings, and the heterotrait–monotrait ratio (HTMT) ratio were used to evaluate the discriminant validity of the measurement model. The square roots of the AVE exceeded the interconstruct correlations, satisfying the Fornell–Larcker criterion; HTMT values were below the threshold of 0.85, further supporting discriminant validity (Table 3). Additionally, all cross‐loadings were higher for their respective constructs than other constructs (Table 4). All outer values VIF were below 3.3, with the highest being 2.570 (POS3), indicating no multicollinearity concerns [51]. The perceived organizational support had a mean score (*M* = 3.85, SD = 0.95), psychological safety had a mean score (*M* = 4.02, SD = 0.63), organizational justice had a mean score (*M* = 3.87, SD = 0.84), and meaningful work had a mean score (*M* = 4.03, SD = 0.72), indicating that the participants had moderately high perceptions of the study variables (Table 2).

**TABLE 3:** Discriminant validity of the constructs.

	MW	OJ	POS	PS
*HTMT criterion*				
MV				
OJ	0.567			
POS	0.592	0.552		
PS	0.476	0.586	0.539	

*Fornell–Larcker criterion*				
MW	0.863			
OJ	0.473	0.825		
POS	0.513	0.482	0.768	
PS	0.393	0.491	0.461	0.754

Abbreviations: MW, meaningful work; OJ, organizational justice; POS, perceived organizational support; PS, psychological safety.

**TABLE 4:** Cross‐loading of the items of the constructs.

	MW	OJ	POS	PS
MW1	**0.838**	0.384	0.421	0.313
MW2	**0.886**	0.456	0.463	0.384
MW3	**0.863**	0.380	0.443	0.317
OJ1	0.385	**0.802**	0.371	0.351
OJ2	0.436	**0.842**	0.425	0.380
OJ3	0.344	**0.852**	0.423	0.450
OJ4	0.404	**0.802**	0.369	0.425
POS1	0.465	0.330	**0.716**	0.319
POS2	0.401	0.344	**0.772**	0.366
POS3	0.420	0.391	**0.822**	0.355
POS4	0.397	0.373	**0.795**	0.388
POS5	0.360	0.401	**0.781**	0.340
POS6	0.376	0.402	**0.783**	0.398
POS7	0.333	0.376	**0.758**	0.377
POS8	0.388	0.342	**0.710**	0.282
PS1	0.297	0.379	0.289	**0.722**
PS2	0.308	0.401	0.390	**0.791**
PS3	0.359	0.354	0.339	**0.752**
PS4	0.264	0.413	0.351	**0.778**
PS5	0.248	0.296	0.368	**0.726**

*Note:* Bolded values indicate the highest factor loading (cross‐loading) of each item on its respective construct.

Abbreviations: MW, meaningful work; OJ, organizational justice; POS, perceived organizational support; PS, psychological safety.

### 3.3. Structural Model Assessment

The structural model was evaluated using several key indices, including the variance inflation factor (VIF), *R* ^2^, adjusted *R* ^2^, *Q* ^2^ predictive relevance, effect size (*f* ^2^), and the standardized root‐mean‐square residual (SRMR) to assess multicollinearity, explanatory power, predictive relevance, and overall model fit. All VIF values were below 3.3, indicating no issues with multicollinearity among constructs [51]. The model explained 31.7% of the variance in psychological safety (*R* ^2^ = 0.317, adjusted *R* ^2^ = 0.311), and 29.5% of the variance in meaningful work (*R* ^2^ = 0.295, adjusted *R* ^2^ = 0.290).

The *f* ^2^ statistic was used to evaluate the effect size of each predictor construct. According to Cohen’s guidelines [52], *f* ^2^ values of 0.35, 0.15, and 0.02 represent large, medium, and small effects, respectively. Perceived organizational support demonstrated a moderate effect on meaningful work (*f* ^2^ = 0.198) and a small‐to‐moderate effect on psychological safety (*f* ^2^ = 0.109). Psychological safety had a small effect on meaningful work (*f* ^2^ = 0.044), and organizational justice exerted a small effect on psychological safety (*f* ^2^ = 0.071).

The interaction term (organizational justice × perceived organizational support) exhibited a negligible effect size (*f* ^2^ = 0.015), falling below the conventional threshold for a small effect. Although the moderation effect reached statistical significance, its practical explanatory impact is limited, and the primary variance in psychological safety is explained by the direct effects of perceived organizational support and organizational justice. Furthermore, *Q* ^2^ values for PS (*Q* ^2^ = 0.292) and meaningful work (*Q* ^2^ = 0.282) exceeded zero, indicating that the model had acceptable predictive relevance. The SRMR value of 0.064 is below the threshold of 0.08, indicating a good overall model fit [53] (Table 5).

**TABLE 5:** Model fit evaluation.

Paths	VIF	*R*‐square	*R*‐square adjusted	*f*‐square	*Q* ^2^ predict	SRMR
OJ ‐> PS	1.689			0.071		0.064
OJ × POS ‐> PS	1.299			0.015		
POS ‐> MW	1.270			0.198		
POS ‐> PS	1.369	0.317	0.311	0.109	0.292	
PS ‐> MW	1.270	0.295	0.290	0.044	0.282	

Abbreviations: MW, meaningful work; OJ, organizational justice; POS, perceived organizational support; PS, psychological safety.

### 3.4. Structural Model Analysis

The structural model’s hypothesized relationships were tested using bootstrapping procedures (5000 resamples), revealing that all direct paths were statistically significant. Perceived organizational support had a positive direct effect on meaningful work (*β* = 0.421, *p* < 0.001, 95% CI [0.310, 0.524]) and psychological safety (*β* = 0.319, *p* < 0.001, 95% CI [0.209, 0.428]). Organizational justice was positively associated with psychological safety (*β* = 0.287, *p* < 0.001, 95% CI [0.163, 0.415]). Psychological safety positively predicted meaningful work (*β* = 0.199, *p* < 0.01, 95% CI [0.067, 0.337]).

The interaction effect between organizational justice and perceived organizational support on psychological safety was negative and statistically significant (*β* = −0.102, *p* < 0.05, 95% CI [−0.192, −0.010]), indicating that higher levels of organizational justice attenuate the positive association between perceived organizational support and psychological safety.

The indirect effect of perceived organizational support on meaningful work via psychological safety was statistically significant (*β* = 0.064, 95% CI [0.019, 0.116]). Similarly, the indirect effect of organizational justice on meaningful work through psychological safety was significant (*β* = 0.057, 95% CI [0.015, 0.119]). Furthermore, the lower bounds of the confidence intervals were close to zero, suggesting that although statistically reliable, the mediation effects are relatively small in practical terms.

**FIGURE 2:**
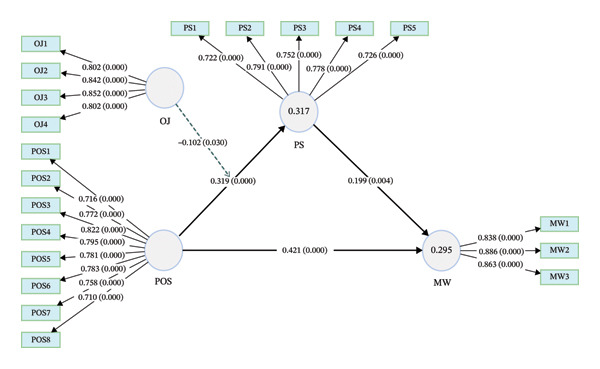
Structural model of the study. Note: path coefficients and associated *p* values are presented along the direct and indirect paths. Outer loadings and corresponding *p* values for each indicator are displayed on the measurement model. Abbreviations: POS: perceived organizational support; OJ: organizational justice; PS: psychological safety; MW: meaningful work.

Moderated mediation was assessed by examining the conditional indirect effects at varying levels of organizational justice. The indirect effect of perceived organizational support on meaningful work via psychological safety was strongest at low levels of organizational justice (−1 SD: *β* = 0.084, *p* < 0.05, 95% CI [0.024, 0.158]), weaker at the mean level (*β* = 0.064, *p* < 0.05, 95% CI [0.019, 0.116]), and weakest at high levels of organizational justice (+1 SD: *β* = 0.043, *p* < 0.05, 95% CI [0.010, 0.086]). Importantly, none of the confidence intervals included zero, supporting the presence of a statistically significant conditional indirect effect across levels of organizational justice. Similarly, the conditional direct effect of perceived organizational support on psychological safety varied as a function of organizational justice. The relationship was stronger at lower levels of organizational justice (−1 SD: *β* = 0.421, *p* < 0.001, 95% CI [0.255, 0.577]), moderate at the mean level (*β* = 0.319, *p* < 0.001, 95% CI [0.209, 0.428]), and weaker at higher levels of organizational justice (+1 SD: *β* = 0.217, *p* < 0.01, 95% CI [0.091, 0.338]). These findings indicate that organizational justice attenuates the positive association between perceived organizational support and psychological safety, thereby weakening the indirect effect of perceived organizational support on meaningful work through psychological safety at higher levels of justice (Table 6, Figure 2).

**TABLE 6:** Bootstrapping results for direct, indirect, and moderated mediation effects.

	*β*	T statistics	95% CI	
			Lower	Upper
*A. Path Coefficients*				
POS ‐> MW	0.421	7.794^∗∗∗^	0.310	0.524
POS ‐> PS	0.319	5.689^∗∗∗^	0.209	0.428
OJ ‐> PS	0.287	4.448^∗∗∗^	0.163	0.415
PS ‐> MW	0.199	2.898^∗∗^	0.067	0.337
OJ × POS ‐> PS	−0.102	2.174^∗^	−0.192	−0.010

*B. Indirect paths*				
OJ ‐> PS ‐> MW	0.057	0.026^∗^	0.015	0.119
POS ‐> PS ‐> MW	0.064	0.025^∗^	0.019	0.116

*C. Conditional indirect*				
POS ‐> PS ‐> MW OJ at +1 SD	0.043	2.218^∗^	0.010	0.086
POS ‐> PS ‐> MW OJ at −1 SD	0.084	2.437^∗^	0.024	0.158
POS ‐> PS ‐> MW OJ at Mean	0.064	2.541^∗^	0.019	0.116

*D. Conditional direct*				
POS ‐> PS OJ at +1 SD	0.217	3.422^∗∗^	0.091	0.338
POS ‐> PS OJ at −1 SD	0.421	5.169^∗∗∗^	0.255	0.577
POS ‐> PS OJ at Mean	0.319	5.689^∗∗∗^	0.209	0.428

Abbreviations: MW, meaningful work; OJ, organizational justice; POS, perceived organizational support; PS, psychological safety.

^∗^ *p* < 0.05.

^∗∗^ *p* < 0.01.

^∗∗∗^ *p* < 0.001.

## 4. Discussion

This study aimed to explore the effects of perceived organizational support, psychological safety, and organizational justice on nurses’ meaningful work experience, using a moderated mediation analysis framework. The model explained 29.5% of the variance in meaningful work and 31.7% of the variance in psychological safety, indicating moderate‐to‐large explanatory power while also highlighting that other important factors remain to be identified.

This study revealed that perceived support from management is positively linked with nurses’ experience of meaningful work, supporting H1. This relationship implies that nurses are more likely to discover purpose and personal meaning in their professional roles when they feel appreciated, supported, and acknowledged by their institutions. These findings were consistent with earlier studies stressing the importance of a supportive work environment in improving meaningful work and professional advantages among nurses [54–56]. Moreover, these findings are consistent with a study by Martela and Riekki [57], which asserts that a workplace environment fulfilling psychological needs like relatedness and competence drives optimal performance and internalized work motivation.

This study found that perceived support from management positively influences psychological safety among nurses, supporting H2. The findings show that when nurses view their organization as appreciating their contributions and prioritizing their well‐being, they are more inclined to feel secure in expressing themselves without fear of shame, rejection, or reprisal.

The current study’s results correspond with previous studies highlighting the essential influence of the organizational environment on psychological outcomes among healthcare professionals. For instance, Nilsson et al. [58] showed that a favorable organizational culture promotes psychological safety and interpersonal trust, both of which are basic components of psychological safety. The present result also concurred with earlier systematic review research showing that healthcare workers who feel their organization values their efforts and are concerned about their wellness have a greater sense of security in expressing themselves [33]. Moreover, the study by Ji and Lee [32] recommended that hospital administrators and nurse managers should promote open communication among nurses by facilitating frequent meetings among healthcare workers to improve psychological safety. This recommendation exemplifies a form of organizational support that aligns with the study’s findings, reinforcing the notion that psychological safety is not an individual trait but a collective organizational outcome [30]. Research by Lackie et al. [45] determined that enablers of psychological safety include prebriefing and debriefing conducted by qualified facilitators, a nonblame culture, and organized, evidence‐based simulation designs. Hierarchical structures within professions, apprehension over errors, and ambiguity were identified as obstacles. From a theoretical perspective, this finding aligns with organizational support theory [22], which posits that employees interpret supportive treatment as indicative of the organization’s intent, thereby enhancing their willingness to engage in interpersonally risky behaviors, such as disclosing mistakes.

This study demonstrated that psychological safety had a direct and significant association with meaningful work among nursing staff, thereby supporting H3. This indicates that when nurses perceive their work environment as psychologically safe, characterized by mutual respect, trust, and the absence of fear of interpersonal risk, they are more likely to experience their work as purposeful and significant. This finding aligned with the existing literature that highlights the value of a psychologically safe climate in healthcare settings. Previous research indicates that when nurses experience psychological safety in the workplace, they are more inclined to communicate openly, thereby increasing job satisfaction, decreasing burnout and turnover intentions, and improving patient safety outcomes [2, 31]. Moreover, a psychologically safe environment enables more social support, thus enhancing commitment [59]. The desire to report safety occurrences among pediatric nurses was predicted by a psychological safe climate [60]. These findings suggest that psychological safety plays a meaningful role in enhancing individual well‐being and professional fulfillment while also encouraging a culture of transparency and accountability. However, given that psychological safety explained only a modest portion of the variance in meaningful work, it is likely one of several contributing factors rather than the primary mechanism. Similarly, Bryant et al. [61] argue that contributing to society as a core element of meaningful work is not solely an other‐oriented construct but also includes self‐oriented fulfillment. From a theoretical perspective, the Situated Expectancy Value theory suggests that people evaluate the meaningfulness of an activity given their expectations about the value of their contributions, the personal costs, and how these outcomes fit their intrinsic expectations [62]. In this light, meaningful experiences for nurses may arise depending on both the social valuation of caregiving and the personal assessment of whether the work fulfills the desired outcomes for both the beneficiary and the self. The environment of psychological safety possibly allows for such appraisals to take place, giving nurses a psychological space to engage in reflection where they align their efforts with their professional calling and expectations.

This research identified psychological safety as a partial mediator in the association between perceived support from management and meaningful work for nursing personnel, hence confirming H4. These findings indicate that a psychologically safe climate serves as a channel through which organizational support enhances the nurses′ perception of meaning at work. This finding is consistent with the prior research, which demonstrated that the perception of organizational support and psychological safety positively and significantly increased work engagement among Chinese nursing professionals during the COVID‐19 pandemic, whereby psychological safety served as the mediator [9]. Moreover, a study by Raineri and Cartes [63] found that psychological safety plays a mediating role between Dark Triad personality traits and performance outcomes in the nursing setting. Therefore, this study demonstrated that Dark Triad traits adversely affect psychological safety and consequently impair the job performance of nursing staff. This point strongly argues in favor of organizational and managerial support for the development of psychological safety to enhance nurses′ performance.

According to a study by Tong [6], the job performance of Chinese nurses was positively related to meaningful work. These studies taken together demonstrate the importance of psychological safety as a route by which organizational support not only fosters meaningful work but also produces better performance results. The JD‐R model helps to provide a useful framework for interpreting the findings, suggesting that job resources, such as perceived support and a psychologically safe climate, are essential for enhancing employee motivation and promoting positive work outcomes [29, 64]. When nurses feel significant organizational support, they create psychological safety in an atmosphere favorable to their sense of purpose, meaning, and involvement in their work. Furthermore, this study will broaden the theoretical framework of Affective Events theory [65], which postulates that positive workplace conditions shape affective experiences affecting work attitudes. This psychological safety could be the affective lens via which the impact of organizational support is changed into significant experiences.

Although psychological safety significantly mediated the associations between perceived organizational support, organizational justice, and meaningful work, the magnitude of the indirect effects was modest. Moreover, the lower bounds of the bootstrapped confidence intervals approached zero, suggesting that the indirect effects, while statistically significant, should be interpreted with caution. This small effect may be due to the predominance of staff nurses (94.6%) in the sample, whose perceptions of meaningful work might be shaped by multiple factors beyond organizational support or justice. Additionally, meaningful work is a complex construct influenced by both personal and organizational variables, suggesting that psychological safety is only one of several mediating pathways. Future research should explore other potential mediators, such as work engagement, professional identity, or affective commitment, which may explain a larger portion of variance in meaningful work.

From a practical standpoint, these small effect sizes indicate that psychological safety explains only a limited portion of the mechanism through which organizational support and justice influence meaningful work. This finding is not entirely surprising given the complexity of meaningful work as a construct, which is shaped by multiple individual, relational, and organizational factors. The results suggest that while psychological safety is a statistically significant mediator, it is likely one of several pathways operating simultaneously. The proximity of the confidence interval lower bounds to zero also raises the possibility that the mediation effects could be unstable across different samples or contexts. This underscores the need for replication studies in diverse healthcare settings to determine whether the observed indirect effects are robust or sample‐dependent. Future research should explore additional mediating processes such as work engagement, professional identity, or affective commitment that may explain a larger proportion of the variance in meaningful work.

For nurse leaders and policymakers, these findings suggest that while fostering psychological safety is valuable, it should not be viewed as the sole or primary mechanism for enhancing meaningful work. Comprehensive strategies that simultaneously address organizational support, justice, psychological safety, and other motivational resources are likely necessary to meaningfully impact nurses′ perceptions of work meaningfulness.

The study found that organizational justice had a direct and significant association with psychological safety, thereby supporting H5. This suggests that fair treatment and transparent procedures within organizations foster surroundings of trust and psychological safety. This finding aligns with previous research indicating that high organizational justice enhances registered nurses′ work‐related outcomes, health, and well‐being [10, 11]. Recent empirical evidence further strengthens this interpretation. For instance, a recent structural equation modeling study demonstrated that organizational justice significantly reduces organizational silence among clinical nurses, thereby encouraging them to express concerns and share knowledge openly [66].

This study found that organizational justice negatively moderates the link between organizational support and psychological safety among nursing staff, thereby confirming H6. Organizational support had a beneficial impact on psychological safety, specifically weakened when nurses believed in high degrees of equality in organizational processes, interactions, and consequences. This pattern is consistent with the negative interaction coefficient, indicating that organizational justice attenuates rather than reverses the positive association between perceived organizational support and psychological safety. In highly just organizations, extra perceived support might be seen as unnecessary or superfluous, thereby reducing its incremental benefit for promoting psychological safety. On the other hand, in environments where justice is seen as low, organizational support might be compensatory and a vital relational resource that boosts employees′ confidence in voicing issues or taking interpersonal risks. This finding challenges the traditional assumption that organizational justice and support are always synergistic in fostering positive outcomes [67]. Instead, it suggests a substitutive interaction between structural and relational resources, wherein the presence of high organizational justice may reduce the relative importance of perceived organizational support for cultivating psychological safety [29, 37]. One plausible explanation is that in environments characterized by high procedural, distributive, and interactional fairness, nurses may perceive organizational systems as sufficiently protective and predictable, thereby diminishing the incremental value of additional supportive gestures from the organization. This aligns with the concept of diminishing marginal returns of job resources under the JD‐R framework [29], when a foundational resource (justice) is already abundant, the added benefit of another resource (support) may plateau.

Alternatively, a ceiling effect may be operative: when psychological safety is already high due to robust organizational justice, there may be limited variance left to be explained by perceived organizational support. This does not imply that support is unimportant in just environments, but rather that its unique contribution may be attenuated when fairness is already well established. It is important to acknowledge that the effect size for the interaction term (*f* ^2^ = 0.015) falls below Cohen’s threshold for a small effect (*f* ^2^ ≥ 0.02). This indicates that while the moderating effect of organizational justice on the relationship perceived organizational support and psychological safety achieved statistical significance, its explanatory power in the model is limited. From a practical standpoint, this suggests that organizational justice explains only a small proportion of the variance in the strength of the relationship between perceived organizational support and psychological safety. Researchers and practitioners should therefore avoid overinterpreting the clinical or managerial significance of this moderation effect. The primary drivers of psychological safety in this study remain the direct effects of perceived organizational support (*f* ^2^ = 0.109, small‐to‐medium) and organizational justice (*f* ^2^ = 0.071, small), which together explain 31.7% of the variance. The interaction, while theoretically interesting, contributes modestly beyond these main effects. Employees may depend more on systematic guarantees than on discretionary support from supervisors or colleagues when organizational systems are seen as fair and transparent. This is in line with COR theory [21], which holds that people give priority to resources depending on need and scarcity; when fairness is absent, support becomes more important. Furthermore, cultural factors especially high‐power distance and uncertainty avoidance, which are common in many Arab healthcare environments, might help to shape this result contextually [68]. In such settings, justice implemented via strict policies might be seen as hierarchical and controlling rather than empowering, thereby lowering workers′ comfort with informal, interpersonal support systems that foster psychological safety.

Interestingly, these outcomes contrast with those in other cultural or organizational settings. For instance, Chang [42] showed that perceived organizational justice positively moderated the link between perceived support from management and citizenship behaviors, therefore strengthening their mutual effects. This difference implies that outcome type, cultural context, or sectoral dynamics could determine the direction and strength of moderation effects. Although organizational citizenship behaviors might thrive under shared views of justice and support, psychological safety being highly relational and emotionally loaded may react differently, particularly in clinical settings where expressing vulnerability has professional risk.

These results highlight the importance of a balanced approach combining relational support techniques with official justice systems. Although developing equitable and open processes is still fundamental to organizational functioning, it should not supplant the relational trust, compassion, and honest communication needed to foster psychological safety. Nursing leaders should be careful not to over‐rely on formalized systems of justice at the expense of emotional intelligence and interpersonal involvement. Particularly in hierarchical healthcare systems, leadership development programs should include training on relational leadership styles, psychological safety facilitation, and cultural competence.

This study confirmed H7 by showing that the degree of organizational justice perceived by nursing staff determines the strength of the indirect association between organizational support and perceptions of meaningful work through psychological safety. Specifically, low perceived justice conditions amplify the indirect effect more than high perceived justice conditions. This implies that in psychologically unsafe or procedurally unjust settings, where relational resources can act as a barrier against the strain and demotivation brought on by systematic injustice, organizational support becomes more important and powerful.

These results provide partial support for the compensatory resource hypothesis under the JD‐R model [29]. In contexts where justice is perceived to be lacking, nurses appear to derive greater value from organizational support, which can mitigate feelings of marginalization or mistrust and reinforce psychological safety. Conversely, in highly just environments, the relative contribution of organizational support to psychological safety diminishes, not because support is unimportant, but because fairness already provides a strong foundation for psychological safety. This pattern is consistent with a resource substitution effect, wherein one resource (justice) can partially substitute for another (support) in fostering psychological safety.

However, given the small effect size of the interaction, it is important to frame this finding as a preliminary observation requiring replication rather than a definitive conclusion. The compensatory role of organizational support may be most relevant in contexts where systemic fairness is inconsistent or perceived as low, conditions that may be more prevalent in some healthcare settings than others. Nurse leaders should therefore prioritize building both just systems and supportive relationships, recognizing that these resources may work synergistically in some contexts and substitutivity in others, depending on the baseline levels of each.

These findings also align with the COR theory [21], which posits that employees become more sensitive to supportive resources when core resources such as fairness, equity, or autonomy are depleted. In such environments, organizational support may serve as a compensatory affirmation of employees’ worth and contribution. On the other hand, the research of Park and Kim [38] carried out in Korean organizational contexts revealed that procedural justice enhanced the indirect link between workplace support and creative behavior by means of psychological empowerment. Their results imply that by strengthening empowerment and creative involvement, fairness increases the motivational effect of support.

### 4.1. Theoretical Contributions

This study extends and refines several organizational behavior theories within the nursing context, particularly in the Saudi Arabian cultural setting. First, the findings advance SET by demonstrating that nurses reciprocate perceived organizational support with engagement and discretionary effort, but the strength of these behaviors is moderated by organizational justice, highlighting relational dynamics in hierarchical and high‐power distance environments common in Arab healthcare contexts. Second, the results provide empirical support for the JD‐R model, showing that job resources such as organizational support and psychological safety interact dynamically with structural resources (justice) to influence meaningful work. This supports the notion of compensatory and substitutive resource effects under varying contextual conditions. Third, the study contributes to COR theory by illustrating that when core resources like organizational justice are abundant, relational resources such as support gain differential significance, which aligns with the theory’s propositions regarding resource prioritization and scarcity. Overall, these contributions validate and extend the applicability of these theoretical frameworks to non‐Western healthcare settings, providing evidence that cultural and contextual factors can shape the mechanisms linking organizational factors to meaningful work.

By explicitly linking these findings to theory, this study not only confirms prior organizational behavior models but also highlights how cultural and contextual factors in Saudi healthcare moderate these theoretical mechanisms. This addresses a critical gap in cross‐cultural validation of organizational behavior theories within nursing research and underscores the importance of considering local norms, hierarchical structures, and relational dynamics when applying established frameworks.

## 5. Limitations

The cross‐sectional design limits the ability to make causal interpretations between the investigated constructs. Furthermore, reliance on self‐reported data may have introduced response biases. The generalizability of the results to other cultural or healthcare institutions may also be constrained by the study setting. The underrepresentation of nurse managers (5.4% of the sample) may further limit applicability to managerial roles. Although the interaction between organizational justice and perceived organizational support on psychological safety was statistically significant, the effect size was negligible (*f* ^2^ = 0.015), indicating limited practical impact. This finding should be interpreted with caution, and future studies with larger samples, longitudinal designs, or experimental interventions may help determine whether this moderating effect is robust and meaningful. The cross‐sectional nature of the study also precludes examination of temporal dynamics, such as whether the effect of organizational justice strengthens or diminishes over time. Finally, some potentially influential factors, such as leadership style, team cohesion, and individual professional identity, were not measured and may have affected the observed relationships. Future research should aim to include these variables and ensure balanced representation across professional levels to enhance generalizability and external validity.

## 6. Implications

The findings of this study offer several actionable implications for nursing practice, management, and policy, particularly within the cultural context of Saudi healthcare settings. Nurse leaders should actively foster supportive and fair work environments by recognizing staff contributions through constructive feedback, tailored professional development opportunities, and acknowledgment of achievements. Promoting psychological safety is critical and can be operationalized through structured team debriefings, nonpunitive error reporting systems, and training programs that encourage open communication and dialog. These strategies enable nurses to voice concerns, strengthen team cohesion, and enhance overall well‐being.

In settings where organizational justice is perceived as low, targeted managerial support becomes especially important. Leaders can provide personalized guidance, engage relationally with staff, and implement transparent policies to mitigate perceptions of unfairness, sustain motivation, and preserve psychological safety. Leadership development programs should incorporate principles of relational leadership, just culture, and psychological safety into both initial and ongoing training to cultivate a supportive and culturally sensitive organizational climate.

At the organizational level, these findings provide evidence‐based guidance for improving nurse retention, engagement, and patient care quality. They also underscore the importance of considering multiple organizational factors collectively rather than in isolation, offering a framework for future research to examine additional mediators, moderators, and contextual variables that influence meaningful work among nurses across diverse healthcare settings.

## 7. Conclusion

This multicenter cross‐sectional study examined how perceived organizational support, organizational justice, and psychological safety influence nurses’ perceptions of meaningful work using a moderated mediation framework. The findings indicate that all three factors are significant positive predictors of meaningful work. Psychological safety plays a small but statistically significant partial mediating role between organizational support and meaningful work, as well as between organizational justice and meaningful work. Organizational justice moderates both the relationship between perceived organizational support and psychological safety and the indirect effect of perceived organizational support on meaningful work via psychological safety. The pattern of interaction suggests a substitutional or diminishing marginal effect, whereby the influence of perceived organizational support weakens as organizational justice increases.

## Author Contributions

Ibrahim Abdullatif Ibrahim is the sole author of this study and was responsible for the conception, design, execution, and interpretation of the research. As the corresponding author, he also drafted and revised the manuscript.

## Funding

No funding was received for this manuscript.

## Disclosure

The author approved the final version for submission.

## Conflicts of Interest

The author declares no conflicts of interest.

## Data Availability

The data that support the findings of this study are available on request from the corresponding author. The data are not publicly available due to privacy or ethical restrictions.
